# Supplementary material for: SARS-CoV-2 Infection Dynamics in the Pittsburgh Zoo Wild Felids with Two Viral Variants (Delta and Alpha) during the 2021–2022 Pandemic in the United States
Source: Animals (Basel). 2023 Oct 4;13(19):3094. doi: 10.3390/ani13193094 (PMC10571823; doi:10.3390/ani13193094)
Supplement: Supplementary file 1 [file animals-13-03094-s001.zip › animals-2597871-supplementary.pdf]

Supplementary Material:

Table S1. Chronology of fecal sampling and viral detection in lions, tigers and lynxes

Lion		Tiger		Lynx	
**Specimen ID**	**RRT-PCR**	**Specimen ID**	**RRT-PCR**	**Specimen ID**	**RRT-PCR**
Lion1 3/31/21	Detected	Tiger 1 12/1/21	Detected	Lynx 1 12/12/21	Detected
Lion1 4/3/21	Detected	Tiger 1 12/4/21	Detected	Lynx 1 12/16/21	Undetermined
Lion1 4/7/21	Detected	Tiger 1 12/8/21	Undetermined	Lynx 1 12/19/21	Undetermined
Lion1 4/7/21	Detected	Tiger 1 12/12/21	Detected	Lynx 1 12/26/21	Undetermined
Lion1 4/11/21	Detected	Tiger 1 12/15/21	Undetermined	Lynx 1 12/30/21	Undetermined
Lion1 4/15/21	Undetermined	Tiger 1 12/19/21	Undetermined	Lynx 1 1/2/22	Undetermined
Lion1 4/19/21	Undetermined	Tiger 1 12/23/21	Undetermined	Lynx 1 1/5/22	Undetermined
Lion1 4/25/21	Undetermined	Tiger 1 12/29/21	Undetermined	Lynx 1 1/9/22	Undetermined
Lion1 4/30/21	Undetermined	Tiger 1 12/31/21	Undetermined	Lynx 1 1/16/22	Undetermined
Lion1 12/24/21	Undetermined	Tiger 1 1/2/22	Undetermined	Lynx 1 1/23/22	Undetermined
Lion1 12/25/21	Undetermined	Tiger 1 1/5/22	Undetermined		
Lion1 12/26/21	Undetermined	Tiger 1 1/9/22	Undetermined	Lynx 2 12/13/21	Undetermined
Lion1 12/31/21	Undetermined	Tiger 1 1/12/22	Undetermined	Lynx 2 12/15/21	Undetermined
Lion1 1/2/22	Undetermined	Tiger 1 1/16/22	Undetermined	Lynx 2 12/19/21	Undetermined
Lion1 1/5/22	Undetermined	Tiger 1 1/23/22	Undetermined	Lynx 2 12/23/21	Undetermined
Lion1 1/11/22	Undetermined			Lynx 2 12/26/21	Undetermined
Lion1 1/18/22	Undetermined	Tiger 2 12/4/21	Detected	Lynx 2 12/30/21	Undetermined
		Tiger 2 12/6/21	Detected	Lynx 2 1/2/22	Undetermined
Lion 2 4/4/21	Undetermined	Tiger 2 12/8/21	Undetermined	Lynx 2 1/5/22	Undetermined
Lion 2 12/21/21	Undetermined	Tiger 2 12/12/21	Detected	Lynx 2 1/9/22	Undetermined
Lion 2 12/23/21	Undetermined	Tiger 2 12/15/21	Undetermined	Lynx 2 1/12/22	Undetermined
Lion 2 1/24/22	Undetermined	Tiger 2 12/19/21	Undetermined	Lynx 2 1/16/22	Undetermined
Lion 2 12/27/21	Undetermined	Tiger 2 12/23/21	Undetermined	Lynx 2 1/23/22	Undetermined
Lion 2 12/29/21	Undetermined	Tiger 2 12/30/21	Undetermined		
Lion 2 1/2/22	Undetermined	Tiger 2 12/31/21	Undetermined	Lynx 3 12/13/21	Undetermined
Lion 2 1/5/22	Undetermined	Tiger 2 1/2/22	Undetermined	Lynx 3 12/15/21	Undetermined
Lion 2 1/10/22	Undetermined	Tiger 2 1/5/22	Undetermined	Lynx 3 12/19/21	Undetermined
Lion 2 1/18/22	Undetermined	Tiger 2 1/9/22	Undetermined	Lynx 3 12/23/21	Undetermined
		Tiger 2 1/12/22	Undetermined	Lynx 3 12/26/21	Undetermined
Lion 3 4/4/21	Undetermined	Tiger 2 1/16/22	Undetermined	Lynx 3 12/30/21	Undetermined
Lion 3 12/21/21	Undetermined	Tiger 2 1/23/22	Undetermined	Lynx 3 1/2/22	Undetermined
Lion 3 12/23/21	Undetermined			Lynx 3 1/5/22	Undetermined
Lion 3 12/29/21	Undetermined			Lynx 3 1/9/22	Undetermined
Lion 3 12/30/21	Undetermined	Tiger 3 12/4/21	Detected	Lynx 3 1/12/22	Undetermined
Lion 3 1/2/22	Undetermined	Tiger 3 12/6/21	Detected	Lynx 3 1/16/22	Undetermined
Lion 3 1/5/21	Undetermined	Tiger 3 12/8/21	Detected	Lynx 3 1/23/22	Undetermined
Lion 3 1/10/22	Undetermined	Tiger 3 12/10/21	Detected		
Lion 3 1/18/22	Undetermined	Tiger 3 12/12/21	Detected	Lynx 4 12/13/21	Undetermined

Lion 3 1/24/22	Undetermined
Lion 4 4/4/21	Undetermined
Lion 4 12/22/21	Undetermined
Lion 4 12/24/21	Undetermined
Lion 4 12/30/21	Undetermined
Lion 4 12/31/21	Undetermined
Lion 4 1/2/22	Undetermined
Lion 4 1/6/22	Undetermined
Lion 4 1/10/22	Undetermined
Lion 4 1/18/22	Undetermined
Lion 4 1/25/22	Undetermined

Lion 5 4/4/21	Detected
Lion 5 4/7/21	Detected
Lion 5 4/11/21	Detected
Lion 5 4/13/21	Detected
Lion 5 4/15/21	Detected
Lion 5 4/19/21	Detected
Lion 5 4/25/21	Detected
Lion 5 4/30/21	Detected
Lion 5 5/4/21	Detected
Lion 5 5/6/21	Detected
Lion 5 5/10/21	Detected
Lion 5 5/13/21	Detected
Lion 5 5/17/21	Undetermined
Lion 5 5/21/21	Undetermined
Lion 5 12/22/21	Undetermined
Lion 5 12/24/21	Undetermined
Lion 5 12/28/21	Undetermined
Lion 5 12/30/21	Undetermined
Lion 5 1/3/22	Undetermined
Lion 5 1/5/22	Undetermined
Lion 5 1/11/22	Undetermined
Lion 5 1/18/22	Undetermined
Lion 5 1/24/22	Undetermined

Lion 6 12/22/21	Undetermined
Lion 6 12/28/21	Undetermined
Lion 6 12/30/21	Undetermined
Lion 6 1/2/22	Undetermined
Lion 6 1/5/22	Undetermined
Lion 6 1/10/22	Undetermined

Tiger 3 12/15/21	Undetermined
Tiger 3 12/19/21	Undetermined
Tiger 3 12/23/21	Undetermined
Tiger 3 12/30/21	Undetermined
Tiger 3 12/31/21	Undetermined
Tiger 3 1/2/22	Undetermined
Tiger 3 1/7/22	Undetermined
Tiger 3 1/16/22	Undetermined
Tiger 3 1/23/22	Undetermined

Tiger 4 12/6/21	Detected
Tiger 4 12/8/21	Detected
Tiger 4 12/12/21	Undetermined
Tiger 4 12/15/21	Undetermined
Tiger 4 12/19/21	Undetermined
Tiger 4 12/22/21	Undetermined
Tiger 4 12/30/21	Detected
Tiger 4 12/31/21	Undetermined
Tiger 4 1/2/22	Undetermined
Tiger 4 1/5/22	Detected
Tiger 4 1/10/22	Detected
Tiger 4 1/12/22	Undetermined
Tiger 4 1/16/22	Undetermined
Tiger 4 1/23/22	Undetermined

Lynx 4 12/15/21	Undetermined
Lynx 4 12/19/21	Undetermined
Lynx 4 12/23/21	Undetermined
Lynx 4 12/26/21	Undetermined
Lynx 4 12/30/21	Undetermined
Lynx 4 1/2/22	Undetermined
Lynx 4 1/5/22	Undetermined
Lynx 4 1/9/22	Undetermined
Lynx 4 1/16/22	Undetermined
Lynx 4 1/23/22	Undetermined

Lynx 5 12/12/21	Undetermined
Lynx 5 12/15/21	Undetermined
Lynx 5 12/19/21	Undetermined
Lynx 5 12/22/21	Undetermined
Lynx 5 12/26/21	Undetermined
Lynx 5 12/29/21	Undetermined
Lynx 5 1/2/22	Undetermined
Lynx 5 1/5/22	Undetermined
Lynx 5 1/9/22	Undetermined
Lynx 5 1/16/22	Undetermined

Lion 6 1/19/22	Undetermined
Lion 6 1/24/22	Undetermined
Lion 6 1/24/22	Undetermined

Table S2. Amino acid substitutions in the SARS-CoV-2 recovered from the lion

Gene	Substitution
**N**	D3L
**N**	R14C
**N**	R203K
**N**	G204R
**N**	S235F
**ORF1a**	I671V
**ORF1a**	T1001I
**ORF1a**	V1291F
**ORF1a**	A1708D
**ORF1a**	I2230T
**ORF1a**	T4175I
**ORF1b**	P314L
**ORF3a**	G188C
**ORF8**	Q27*
**ORF8**	R52I
**ORF8**	Y73C
**S**	N501Y
**S**	A570D
**S**	D614G
**S**	P681H
**S**	T716I
**S**	S982A
**S**	D1118H

Table S3. Amino acid substitutions in the SARS-CoV-2 recovered from the tiger

Gene	Substitution
**M**	I82T
**N**	D63G
**N**	R203M
**N**	G215C
**N**	D377Y
**N**	A414S
**ORF1a**	T403I
**ORF1a**	E767K
**ORF1a**	D1228G
**ORF1a**	A1306S
**ORF1a**	E1724D
**ORF1a**	P2046L
**ORF1a**	P2287S
**ORF1a**	V2930L
**ORF1a**	A3143V
**ORF1a**	T3255I
**ORF1a**	T3646A
**ORF1a**	C3766F
**ORF1a**	S4398L
**ORF1b**	P314L
**ORF1b**	G662S
**ORF1b**	P1000L
**ORF1b**	A1291S
**ORF1b**	A1918V
**ORF3a**	S26L
**ORF3a**	S171L
**ORF3a**	E239Q
**ORF7a**	V82A
**ORF7a**	T120I
**ORF7b**	T40I
**ORF9b**	T60A
**S**	T19R
**S**	G142D
**S**	R158G
**S**	L452R
**S**	D614G
**S**	P681R
**S**	D950N
